# Haemodynamic and functional consequences of the iatrogenic atrial septal defect following Mitraclip therapy

**DOI:** 10.1007/s12471-016-0928-1

**Published:** 2016-11-28

**Authors:** E. A. Hart, K. Zwart, A. J. Teske, M. Voskuil, P. R. Stella, S. A. J. Chamuleau, A. O. Kraaijeveld

**Affiliations:** Department of Cardiology, division Heart and Lungs, University Medical Center Utrecht, Utrecht, The Netherlands

**Keywords:** MitraClip, Iatrogenic atrial septal defect, Mitral regurgitation

## Abstract

Percutaneous MitraClip placement for treatment of severe mitral regurgitation in high surgical risk patients is a commonly performed procedure and requires a transseptal puncture to reach the left atrium. The resulting iatrogenic atrial septal defect (iASD) is not routinely closed, yet the haemodynamic and functional consequences of a persisting defect are not fully understood. Despite positive effects such as acute left atrial pressure relief, persisting iASDs are associated with negative consequences, namely significant bidirectional shunting and subsequent worse clinical outcome. Percutaneous closure of the iASD may therefore be desirable in selected cases. In this review we discuss the available literature on this matter.

## Introduction

Percutaneous mitral valve repair for the treatment of severe mitral regurgitation has become a safe and widely used alternative for mitral valve surgery in high-risk surgical patients [1–4]. Although repair with the MitraClip (Abbott Vascular Structural, Menlo Park, California, USA) is less effective at reducing mitral regurgitation (MR) compared with conventional surgery, the procedure is associated with superior safety and similar improvements in clinical outcome with respect to quality of life, heart failure status and left ventricular function [5].

**Fig. 1:**
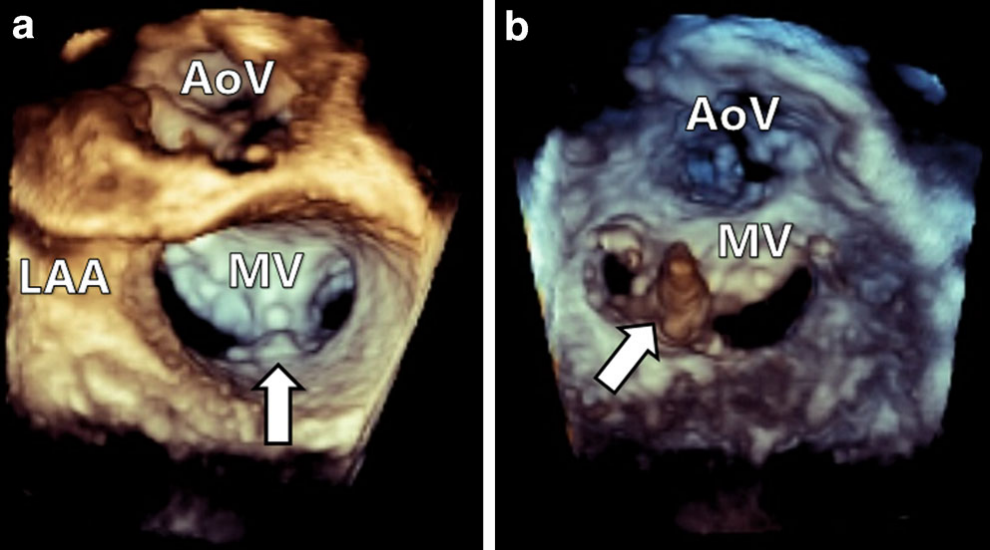
3D atrial view (**a**) and 3D ventricular view (**b**) of MitraClip in situ (*arrows*)

The MitraClip guiding catheter (24 French) is commonly introduced via the right femoral vein and requires a transseptal puncture to access the left atrium, leaving an iatrogenic atrial septal defect (iASD). Manipulation of the catheter allows the system to grip the mitral valve leaflets, subsequently effectively reducing the regurgitant orifice ([6]; Fig. 1).

**Fig. 2:**
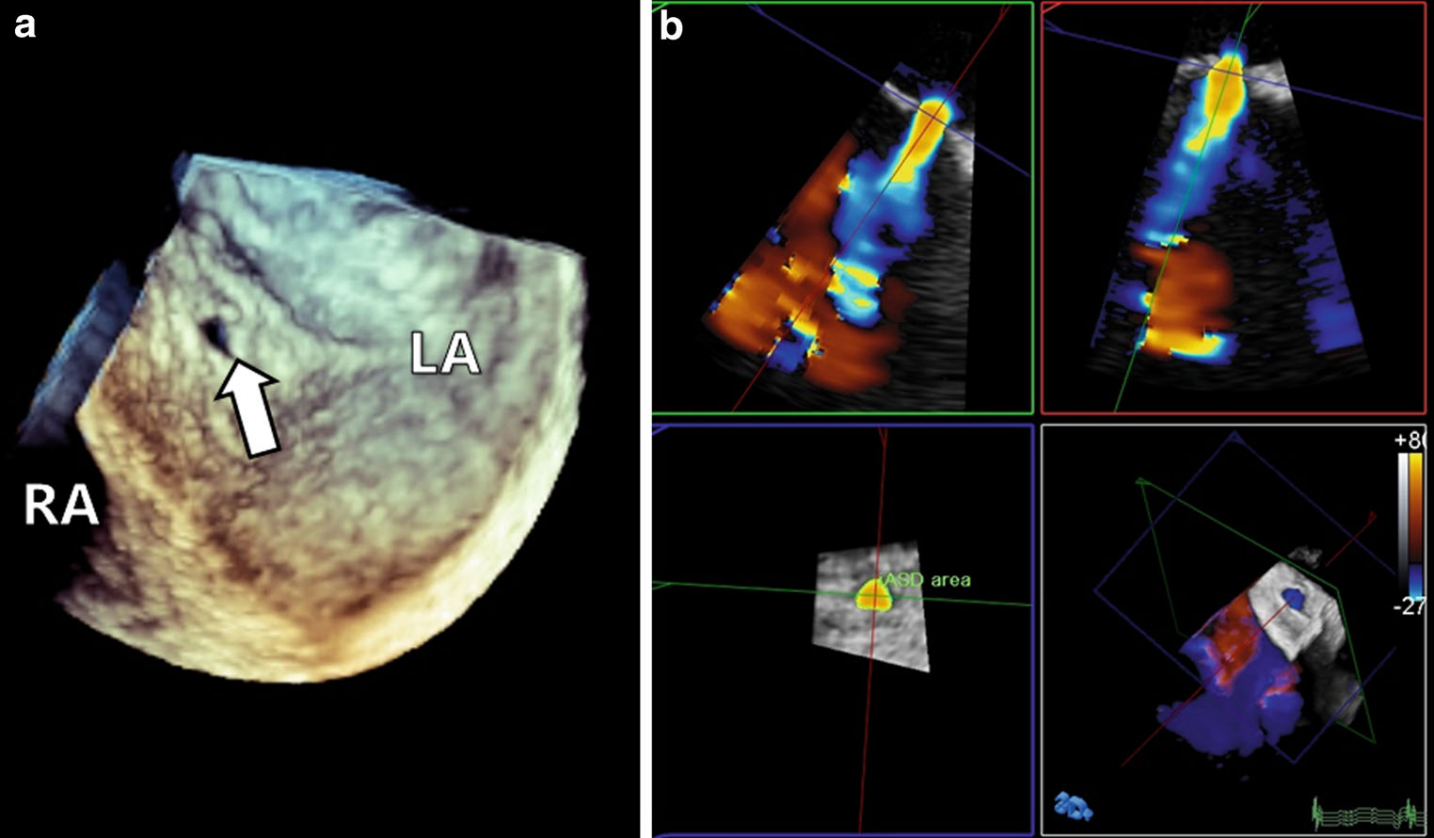
3D view of iatrogenic atrial septum defect (**a**) and display of shunt using colour Doppler (**b**)

A complete overview of the MitraClip procedure is described by Feldman et al. [1]. Other percutaneous procedures (7–14 French) requiring transseptal punctures, such as pulmonary vein isolation, left atrial appendage closure, and percutaneous balloon mitral valvuloplasty likewise result in iASD and persistent shunting has been reported [7–9]. The haemodynamic consequences in these procedures are thought to be minimal and therefore closure of the iASD is not routinely performed. As iASD size increases exponentially with catheter size[10], the iASD following the MitraClip procedure (Fig. 2) might induce haemodynamically significant shunting, although little is known on this subject.

For example, atrial septum defects with long-term left-to-right shunting are associated with right ventricular (RV) dilatation, ultimately leading to RV failure, pulmonary hypertension, and arrhythmias [11–14]. In this review we discuss the aspects of the iASD after MitraClip placement and focus on 1) the prevalence, 2) haemodynamic consequences, 3) case report publications, and finally 4) individual risk assessment.

A PubMed search was performed on 12 May 2016, identifying studies related to iASD following MitraClip therapy. Search terms used were: ((atrial septal defect) OR ASD OR (septal defect)) AND (mitraclip OR (mitral clipping) OR (mitral valve clips) OR (mitral valve repair) OR (mitral repair)). The search yielded 869 hits. We included relevant case reports, articles reporting on the prevalence of iASD following the MitraClip procedure, and articles discussing haemodynamic consequences of the iASD. After cross-checking references we found four papers reporting on the prevalence[13, 15–17], four papers assessing the haemodynamic consequences[13, 15, 16, 18], and four papers reporting on a total of seven case reports [16, 19–21].

## Prevalence

The prevalence of the iASD has been investigated in several studies (Table 1).

**Table 1:** Prevalence of iASD following MitraClip therapy

Study	Method	*N*	Prevalence of iASD		
			1 month	6 months	12 months
Smith et al. 2012[15]	TTE	30	43%	27%	27%
Ussia et al. 2014[16]	TTE	28	81%	n/a	n/a
Saitoh et al. 2012[17]	TEE	11	82%	n/a	n/a
Schueler et al. 2015[13]	TEE	66	n/a	50%	n/a

*iASD* iatrogenic atrial septal defect, *TTE* transthoracic echocardiography, *TEE* transoesopheagal echocardiography

Smith et al. reported a 1-month prevalence of 43% (average diameter 6.0 mm ± 2.3 mm), and 27% (6.6 mm ± 3.1 mm) at 12 months, identified using colour flow Doppler in the apical four chamber or subcostal view on transthoracic echocardiography (TTE) [15]. Ussia et al. (TTE) and Saitoh et al. (transoesophageal echocardiography (TEE)) recorded a significantly higher prevalence of 81% (average diameter 4.5 mm ± 3.1 mm) and 82%, respectively, after one month while Schueler et al. observed a 50% prevalence using TEE and colour Doppler at 6 months with a maximal diameter of 4.3 mm ± 1.7 mm and minimal diameter of 3.8 mm ± 2.1 mm [13, 16, 17].

Various studies have reported on iASD prevalence following non-MitraClip procedures (i. e. ablation procedures[7, 22–24], left atrial appendage closure [25], and percutaneous balloon mitral valvuloplasty [9, 26–29]). McGinty et al. grouped iASD incidence from these studies and found an average iASD prevalence of >35% immediately following the procedure, 20% at 1–6 month follow-up, and >10% at 6 months [30]. A direct correlation between catheter size, iASD diameter, and prevalence[10, 16] may explain the higher iASD prevalence following MitraClip placement compared with non-MitraClip procedures.

Predisposing factors for the long-term prevalence of iASDs have been suggested and include residual high transmitral gradient, left ventricular (LV) hypertrophy, increased left atrial (LA) pressure from residual MR (MR grade 2.7 ± 1.1 for iASD versus 1.5 ± 0.9 for non-iASD)[15], duration of the procedure[7, 15, 16, 27], and mitral valve calcification [9, 28].

## Haemodynamic consequences

We identified four studies assessing haemodynamic and functional consequences of the iASD following MitraClip placement (Table 2).

**Table 2:** Haemodynamic and functional consequences of iASD

Study	*N*	Effect	Conclusion
Hoffman et al. 2014[18]	28	Positive	Immediate volume and pressure relief left atrium
Smith et al. 2012[15]	30	Neutral	iASDs are not haemodynamically significant
Ussia et al. 2014[16]	28	Negative	Three (11%) patients developed negative haemodynamic consequences, requiring closure of the iASD
Schueler et al. 2015[13]	66	Negative	Persistent iASDs are associated with worse clinical outcome and increased mortality rates

*iASD* iatrogenic atrial septal defect

Hoffman et al. included 28 high-risk or inoperable patients with symptomatic mitral regurgitation [18]. TEE was used to measure the velocity-time integral across the iASD and the iASD area (0.19 cm^2^ ± 0.05) immediately following withdrawal of the guiding catheter, which allowed the calculation of the shunt volume (14 ± 6 ml/beat). Invasive LA pressure measurements showed a reduction from 17 ± 8 mm Hg to 15 ± 8 mm Hg upon catheter withdrawal, contributing to LV preload reduction. The authors concluded that the iASD resulted in immediate volume and pressure relief of the left atrium. Unless the patient suffers from pulmonary hypertension this effect may prove to be beneficial, although long-term effects were not assessed in this study.

Smith et al. reviewed the echocardiographic features and predictors of iASD following MitraClip placement in 30 patients [15]. Using TTE, RV size, LA volume and tricuspid/mitral regurgitation were quantified in both iASD and non-iASD patients at 12-month follow-up. No significant differences were found in right heart mid-ventricular diameter or LA volume, although the prevalence of iASD was associated with a relatively high LA volume index (51.4 ± 14 ml) at 12 months. Compared with non-iASD patients, there was a significantly higher average tricuspid regurgitation grade (1.1 ± 0.6 for non-iASD vs. 2.1 ± 1.8 for iASD) and persistent MR grade (1.5 ± 0.9 for non-ASD vs. 2.7 ± 1.1 for iASD) at 12 months. In conclusion, the authors stated that although the iASD does not appear to be haemodynamically significant, it is associated with relatively high LA pressures, suggestive of a causative role of high LA pressures in the persistence of the iASD.

Ussia et al. assessed the iASD in 28 patients following MitraClip repair [16]. Three patients deteriorated haemodynamically, two of which immediately following the procedure, and required iASD closure after which immediate improvement occurred; these cases are described later in this review. The authors suggested the following three steps to be taken to aid in the early recognition of potential iASD complications: 1) 3D-TEE assessment of iASD shape and diameter (laceration or diameter >8 mm may impair spontaneous closure), 2) post-procedural atrial pressure and O_2_ saturation measurement through Swan-Ganz catheterisation to assess shunt significance, and 3) detection of new supraventricular arrhythmias which may indicate haemodynamic deterioration.

The study performed by Schueler et al. prospectively followed 66 patients for 6 months following MitraClip implantation and compared clinical outcomes between persistent iASD patients (*n* = 33) vs. non-iASD patients (*n* = 33) at follow-up [13]. Apart from a slightly larger basal (5.1 ± 0.8 cm for iASD vs. 4.6 ± 0.8 cm for non-iASD *p* = 0.01) and mid-ventricular (3.7 ± 0.8 cm for iASD vs. 3.3 ± 0.7 cm for non-iASD *p* = 0.03) RV diameter, no differences in baseline criteria between the two groups were observed. During follow-up, the iASD group showed worsening of systolic pulmonary artery pressure (PAP) post clip placement (+1.6 mm Hg vs. −10.9 mm Hg for non-iASD, *p* = 0.02) and a larger portion of patients remained within New York Heart Association (NYHA) class >II heart failure. At follow-up, the iASD group showed a significant reduction in LA volume (162.5 ± 63.3 ml to 139.1 ± 47.6 ml) suggestive of a positive influence of the iASD. However, the iASD group presented with higher levels of N‑terminal pro-brain natriuretic peptide (6667.3 ± 7363.9 ng/dl vs. 4835.9 ± 6681.7 ng/dl for non-iASD, *p* = 0.05) and less improvement in the 6‑minute walk test (20.8 ± 107.4 m vs. 114.6 ± 116.4 m, *p* = 0.001). Furthermore, a persisting iASD was associated with a higher all-cause mortality rate at 6 months (16.6% vs. 3.3%, *p* = 0.05). The cause of death was not reported and numbers were relatively small, these findings should therefore be interpreted with caution. Furthermore, as stated by the authors, it is unknown whether a persisting iASD is the cause or effect of worse clinical outcomes. Future studies should investigate the exact mechanism behind iASD persistence and subsequently analyse long-term clinical outcomes.

## Case studies

We identified four case reports describing a total of seven patients. Huntgeburth et al. described two patients older than 70 years with functional MR grade 4+, severely impaired ejection fraction (30–35%), renal impairment and pulmonary disease [19]. These initially stable patients rapidly deteriorated following successful MitraClip placement. Echocardiographic imaging revealed a bidirectional shunt, predominantly left-to-right, in the first patient. Acute right heart failure and cardiogenic shock prompted iASD closure with an Amplatzer device, after which the patient showed immediate haemodynamic improvement. The second patient presented with impaired LV function (left ventricular ejection fraction (LVEF) of 30%), chronic obstructive pulmonary disease, and an enlarged right heart chamber with compression of the LV. The patient experienced severe right-to-left shunting with a subsequent decline in oxygen saturation in merely hours after the procedure, which resolved after percutaneous iASD closure. The authors highlighted the need for post-interventional screening for relevant shunt volumes and 24-hour availability for emergency iASD closure.

Ussia et al. described three patients with NYHA class III/IV heart failure undergoing MitraClip placement experiencing haemodynamic deterioration [16]. All three patients showed a final MR grade reduction of at least 2 and iASD size ranged from 0.65 to 1 cm. One patient showed significant left-to-right shunting eventually leading to the development of right-sided heart failure (15 days post-procedure). The second patient presented with severely impaired LVEF (20%) and a systolic PAP of 30 mm Hg. Following withdrawal of the catheter the patient immediately developed bronchospasm and pulmonary hypertension with a systolic PAP of 50 mm Hg and a bidirectional shunt was detected. The third patient presented with pulmonary hypertension (systolic PAP 65 mm Hg) and after catheter withdrawal the systolic pulmonary pressure increased, right-sided chamber enlargement was observed and a left-to-right shunt was detected, eventually leading to cardiorespiratory arrest. All three cases were resolved through percutaneous closure with either an Amplatzer or Figulla Occluder.

Likewise, Losi et al. reported on a 54-year-old patient with chronic heart failure (NYHA class IV), poor LVEF, increased systolic PAP (70 mm Hg), and high grade functional MR [20]. Immediately following the procedure the patient experienced a significant drop in the partial pressure arterial oxygen/fraction of inspired oxygen (PaO_2_/FiO_2_) ratio from 160 to 80, indicative of right-to-left shunting. Closure of the septal defect with an Amplatzer device normalised the PaO_2_/FiO_2_ ratio to 180. The authors suggested a possible role for the PaO_2_/FiO_2_ ratio as guidance in identifying a clinically significant shunt.

Finally, Chandraprakasam and Satpathy described the case of a 88-year-old female with NYHA class IV heart failure, right ventricular systolic pressure of 60 mm Hg, and severe MR undergoing successful MitraClip placement [21]. Following the procedure she experienced persistent hypoxaemia (O_2_ saturation 85%) despite adequate end-expiratory values and a FiO_2_ of 100%. TEE revealed a predominantly right-to-left shunt. The defect was closed with an Amplatzer device after which the patient improved significantly.

Although rare, these seven case reports highlight possible consequences of a persisting iASD. The need for iASD closure, and in which patients this may be beneficial, remains an important discussion. In four of these cases, right-to-left shunting was detected, indicative of significantly elevated right heart pressures. Prophylactic closure of the defect should be considered in this patient group, although the suitability of these patients for the MitraClip procedure may altogether be questioned.

## Risk assessment

Complications related to the iASD are relatively rare and prophylactic closure of the defect in all patients may therefore not be a desirable addition to clinical care. Closure of the defect is a permanent solution and prevents any future transseptal procedures. Furthermore, in the acute phase following clip implantation, the iASD may initiate left-to-right shunting which may reduce LA pressure and LV preload, a potentially beneficial effect. Indeed the effect of deliberate transcatheter creation of an intracardiac shunt has recently been analysed in non-MitraClip heart failure patients. The resulting left-to-right shunt in patients with preserved [31] or reduced [32] LVEF showed a marked decrease in pulmonary capillary wedge pressure in both groups and an improvement in NYHA heart failure class and quality of life score [32].

On the other hand, a careful selection of at risk patients and subsequent closure of the defect in these patients may be beneficial. Patients with marked pulmonary hypertension with right-to-left shunting are at risk of developing significant hypoxia and iASD closure should be considered. Furthermore, patients with poor RV function, pulmonary hypertension and/or significant left-to-right shunting may profit from closure as well. Exact cut-off values for pulmonary hypertension, RV function and left-to-right shunting volume indicating the need for iASD closure are unknown but are of interest for future research. As suggested by others, post-procedural haemodynamic monitoring by right heart catheterisation, TEE assessment of shunting and defect size[16], and transient balloon occlusion[14] may aid in selecting these at risk patients.

## Conclusion

MitraClip patients are most often high-risk surgical patients and represent a unique and relatively vulnerable population. Although closure of the iASD following catheter-based interventions is not routinely performed, the possible consequences of iASD following MitraClip therapy are still not fully understood. Studies have reported both positive and negative effects of iASD. Therefore, prospective studies analysing the role of iASD closure are needed to fully understand the mechanism and impact of the iASD and to identify patients in which closure may be beneficial.
